# Supplementary material for: Optical vortex knots – one photon at a time
Source: Sci Rep. 2016 Apr 18;6:24463. doi: 10.1038/srep24463 (PMC4834479; doi:10.1038/srep24463)

Optical vortex knots – one photon at a time: Supplementary information

Sebastien J. Tempone-Wiltshire[†*] Shaun P. Johnstone[*] Kristian Helmerson[*]

March 8, 2016

1 Supplementary methods: Altering the geometry of a knotted optical field

To generate an isolated knotted vortex structure within an optical field, we utilise the construction of Ref. 15 in the main text , where the knot is first generated as a nodal structure within a complex scalar field and then approximated via a paraxial optical field. This construction relies upon the recognition that the panoramic view of any torus knot, a knot which can exist entirely upon the surface of a toroid, can be viewed as a braid with periodic boundary conditions, the periodic boundary conditions guaranteeing the closure of the knot. Once the mathematical form of the braid describing the particular knot of interest is calculated, the braid can then be mapped onto the surface of a torus via a Milnor map, which generates the desired knot.

To generate a knotted vortex structure via the above construction, the braids which form the knotted structure must be topological defects of the complex scalar field. For a trefoil knot, as used for the main result of this paper, a two stranded braid with three crossings is required. Following Padgett's convention we parameterise the locations of these strands, the nodes of the complex field, via a parameter $h \in [0, 1]$, which can be viewed as the height of a cylinder in which the strands are drawn upon and map directly to the azimuthal angle of the torus. The complex polynomial describing the 2 stranded braid can thus be written as the product of two complex polynomials of order 1, i.e.

$$p_h(\mu) = \prod_{j=1}^{2}(\mu - s_j(h)). \tag{1}$$

Here μ is a complex variable and $s_j(h)$ denotes the location of a strand in the complex plane for a given value of the height parameter of the cylinder.

It is here we make our extension to the original construction, which allows the geometry of the knot to be altered without changing the topology. In the original construction for a trefoil knot the nodal strands follow a helical trajectory, and thus each strands projection onto the bottom of the cylinder traces out a circle one and a half times with each strand's position within a given plane being π out of phase with respect to the other strand. We extend this construction by forcing the projection of the braids trajectory to follow an elliptical path instead of a circular path, i.e the locations of the strands is described by

$$s(h) = \frac{a(1-b^2)}{1 + b\cos(\frac{3\pi h}{2})} e^{i\frac{3\pi h}{2}}, \tag{2}$$

instead of the form in Ref. 15,

$$s(h) = e^{i\frac{3\pi h}{2}}. \tag{3}$$

This introduces two new parameters into the construction, a, the major axis of the ellipse, and b, the eccentricity of the ellipse. The extension provided here does not change the topology of the resulting complex scalar field, rather only the geometry, as the extension can be viewed as a geometric transformation of the cylinder the braids are embedded within. The resulting complex scalar field describing the location of the vortices within the cylinder is thus given by

$$p_h(\mu) = \mu^2 - \left[\frac{a(1-b^2)}{1 + b\cos(\frac{3\pi h}{2})}\right]^2 e^{i3\pi h}. \tag{4}$$

This complex scalar field is then mapped from the elliptical cylinder to an elliptical torus via a Milnor map, generating a 3-dimensional complex scalar field containing a knotted nodal line with the topology of a trefoil knot. Note that this 3-dimensional complex scalar field need not obey any paraxial equations with regards to evolution of the complex field, and thus may not necessarily be realisable with light. To recreate this resultant nodal structure in an optical field, a transverse slice of the complex scalar field is taken and recreated in the paraxial approximation through a superposition of Laguerre–Gaussian (LG) modes.

[*]School of Physics and Astronomy, Monash University, Victoria 3800, Australia, [†]sebastien.tempone-wiltshire@monash.edu

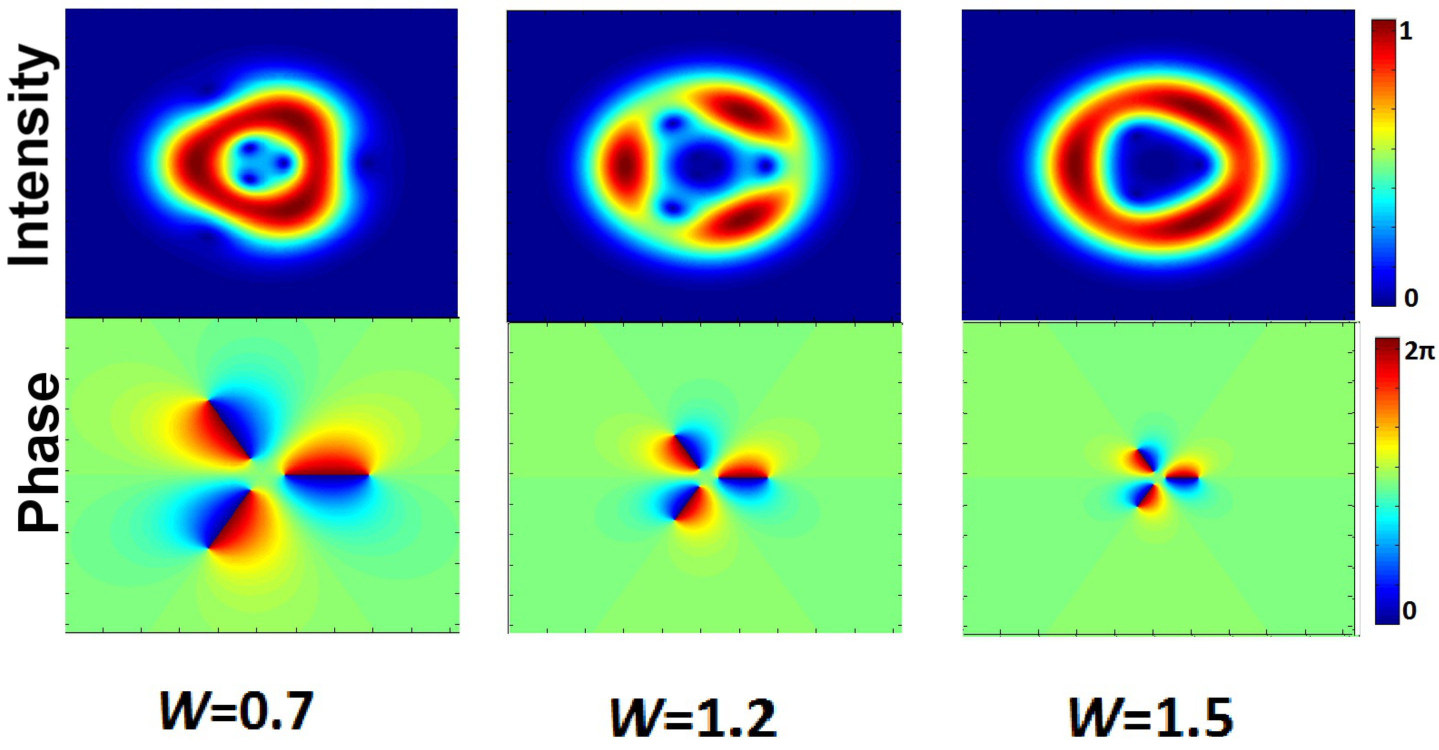

Figure 1: **The effect of varying W**. False colour images of the (normalised) intensity and phase of a transverse slice of the knotted optical field for various values of the 'waist' parameter, W. This parameter is seen to 'scale' the vortex structure, decreasing both the inner vortex separation and the separation between the inner and outer vortices as W is increased.

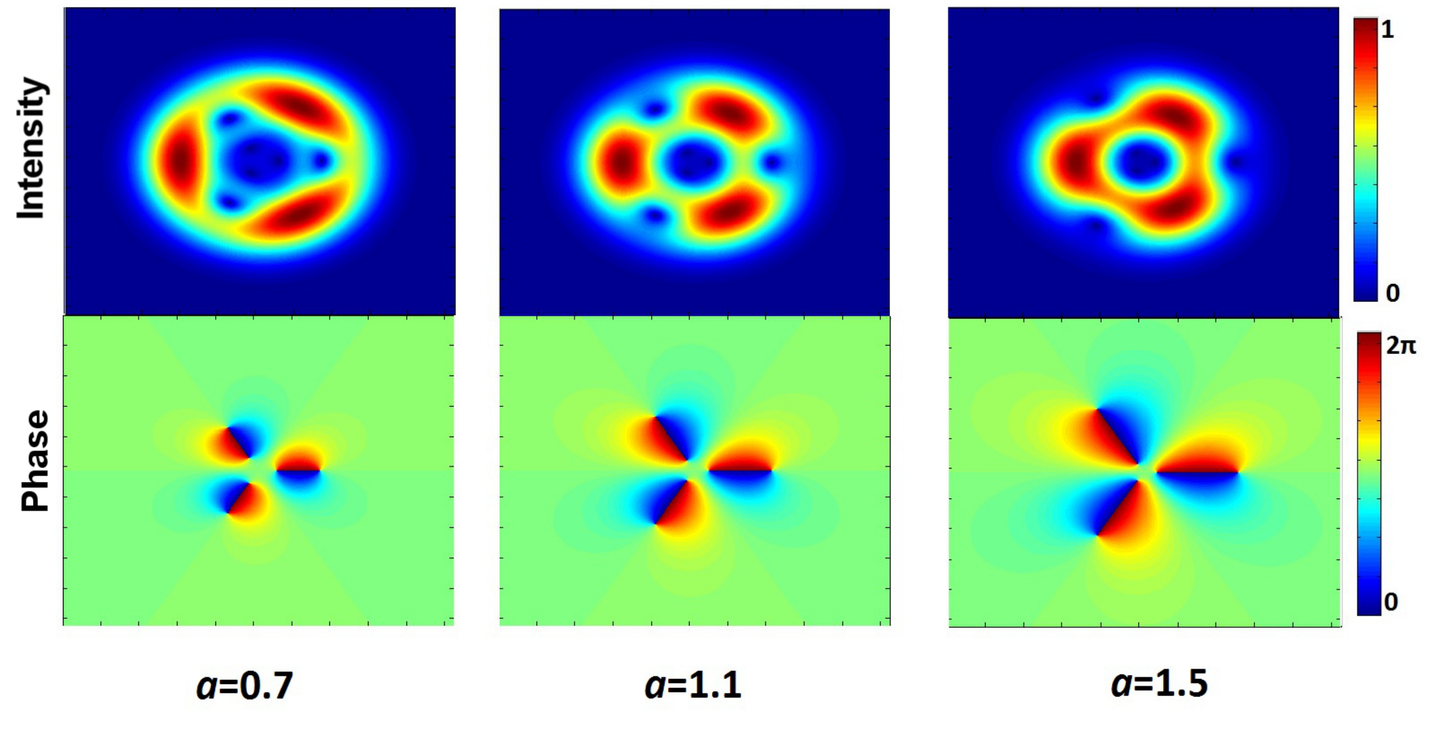

Figure 2: **The effect of varying a.** The effect of varying the length of the parameter a, increasing from left to right, on both the intensity and phase of the knotted optical field. The parameter a is seen to greatly increase the separation between the inner and outer vortices while only slightly decreasing the separation between the inner vortices alone.

2 Supplementary discussion: The effect of introducing the two new parameters

The construction of Ref. 15 involves approximating the complex knotted field by LG modes, introducing one free parameter, the 'waist' of the LG modes with respect to the cylinder (or toroid) size. The structure of the phase of the optical field, shown

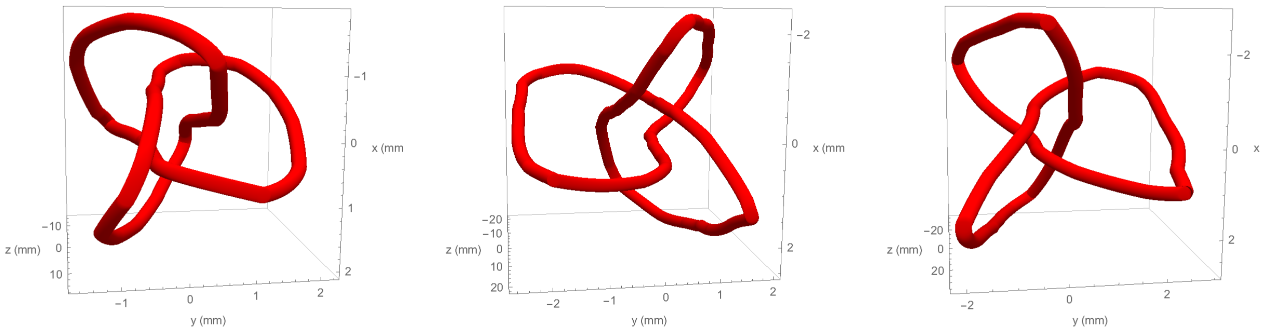

(a) (b) (c)

Figure 3: **Three experimentally realised knotted nodal structures. a-c,** Shown are three knotted nodal structures, each with a different length along the propagation direction (z) due to the different values of the parameter a used to form the optical field. For each knot the parameter W was keep constant at a value of 1.2, while the values of a were 0.7, 1 and 1.3 for **a**, **b** and **c**, respectively. The overall change in length of the knot is ≈ 3

on the bottom half of Fig. 1 clearly shows that altering the 'waist' parameter, W, of the construction simply scales the entire structure, decreasing both the distance between the inner vortices, and the distance between the inner and outer vortices at the same rate, with increasing values of W. There are both positive and negative aspects to changing this parameter. Smaller nodal structures (such as when W=1.5 in Fig. 1) decrease the chance of reconnection events from vortices outside the optical field and are therefore more likely to retain the desired knotted nodal topology; however, the structures also then lie in regions of near darkness and can be hard to locate interferometrically. On the other hand when W is small the nodal structure are much larger and the vortices are well separated allowing their locations to be easily identified; however, the chances of reconnection events due to vortices outside the optical field is increased and the nodal structure is less likely to be retained.

In Fig. 2 the effect of changing one of our new introduced parameters, a, is shown. Again, the effect of this parameter upon the resultant field is most clearly seen in the phase profile of the optical field, with increasing values of a seen to increase the separation of the vortices. The distinction to make here is that although the parameter W can be used to increase the separation of the inner and outer vortices, it does so by also increasing the separation distance of the inner vortices, i.e. scaling the whole structure, whereas our parameter a allows the separation of the inner and outer vortices to be altered without changing the separation of the inner vortices.

Simulations show that both of these parameters affect the resultant length of the knotted structure, with the length increasing with increasing values of a, and descreasing with increasing values of W. Using these two parameters, we can adjust the aspect ratio of the resultant knotted optical field, defined as the ratio of the length of the nodal structure to the waist of the field in which it is embedded, while also ensuring the vortices are still identifiable and that the knotted nodal structure remains. In Fig. 3 we show three experimentally realised knotted optical vortex fields, all of which have different lengths of the knotted nodal structure. The lengths varying by a factor of $\approx$3 from the shortest to the longest knot. These three structures are produced and identified with the optical set up described in the main text, however, each knotted field corresponds to a different value of the parameter a, 0.7, 1 and 1.3, while the parameters b and W are kept constant at values of 0 and 1.2, respectively.

It is important to note that not all values of the introduced parameters necessarily generate a knotted nodal structure in the optical field. This is because these introduced parameters modify the nodal structure of the entire complex scalar field that is then approximated by a paraxial optical field when decomposed into the LG basis. A clear example of this is seen through the eccentricity parameter we introduced, b, which breaks the three-fold symmetry of the knotted complex field, readily destroying the topology of the vortex structure in the optical field. As the parameter b readily destroyed the knotted topology and was found to only have a small affect upon the length of the knot when the topology was not destroyed, this was not investigated further.

3 Supplementary data

Page 5 shows the full resolution hologram displayed upon the SLM to generate the knotted topological structure in the optical field, and to also generate a reference Gaussian beam to interferometrically measure the vortex structure of the knotted field. Page 6 shows the two full resolution holograms displayed upon the SLM side by side to generate the two separate beams comprising different LG modes which are then recombined to form the knotted optical field.

4 Supplementary video 1; Imaging an optical knot – one photon at a time

This video shows how a single high contrast measurement of the optical field is generated by summing together thousands of single photon measurements of the optical field.

5 Supplementary video 2; The measured isolated optical vortex knot

This video offers a rotating view of the 3-dimensional vortex structure, verifying the knotted topology of the vortices.

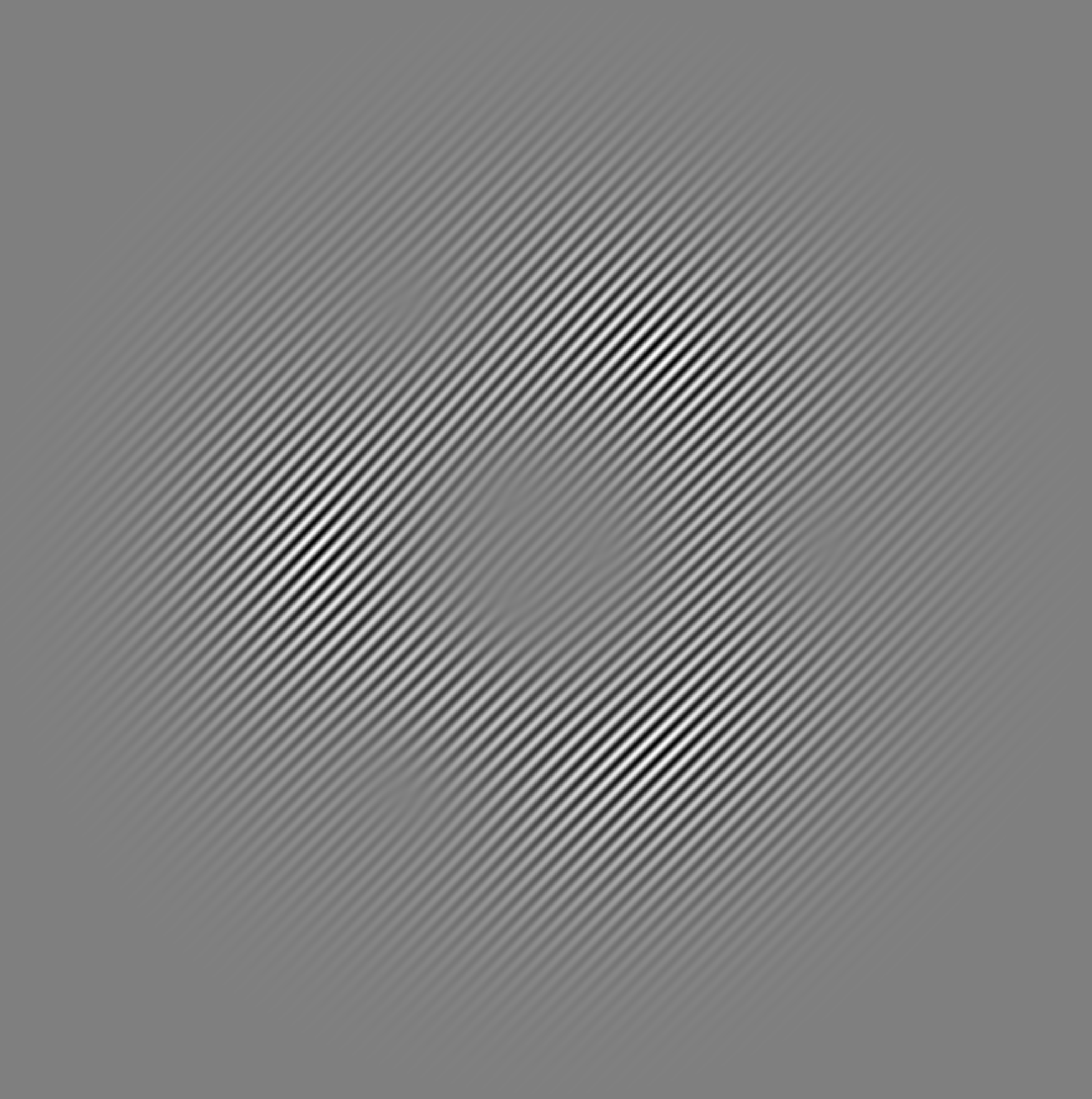

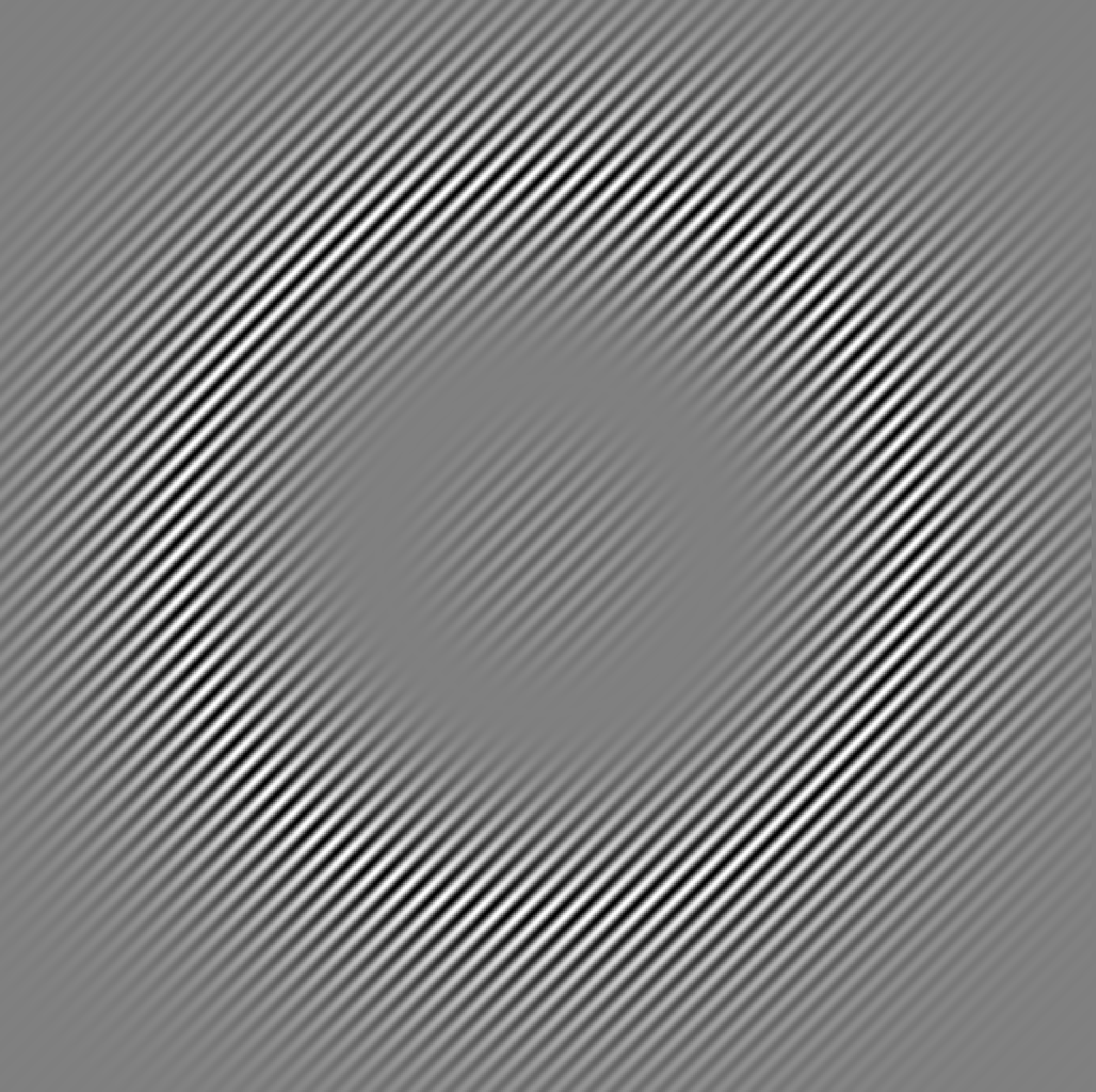
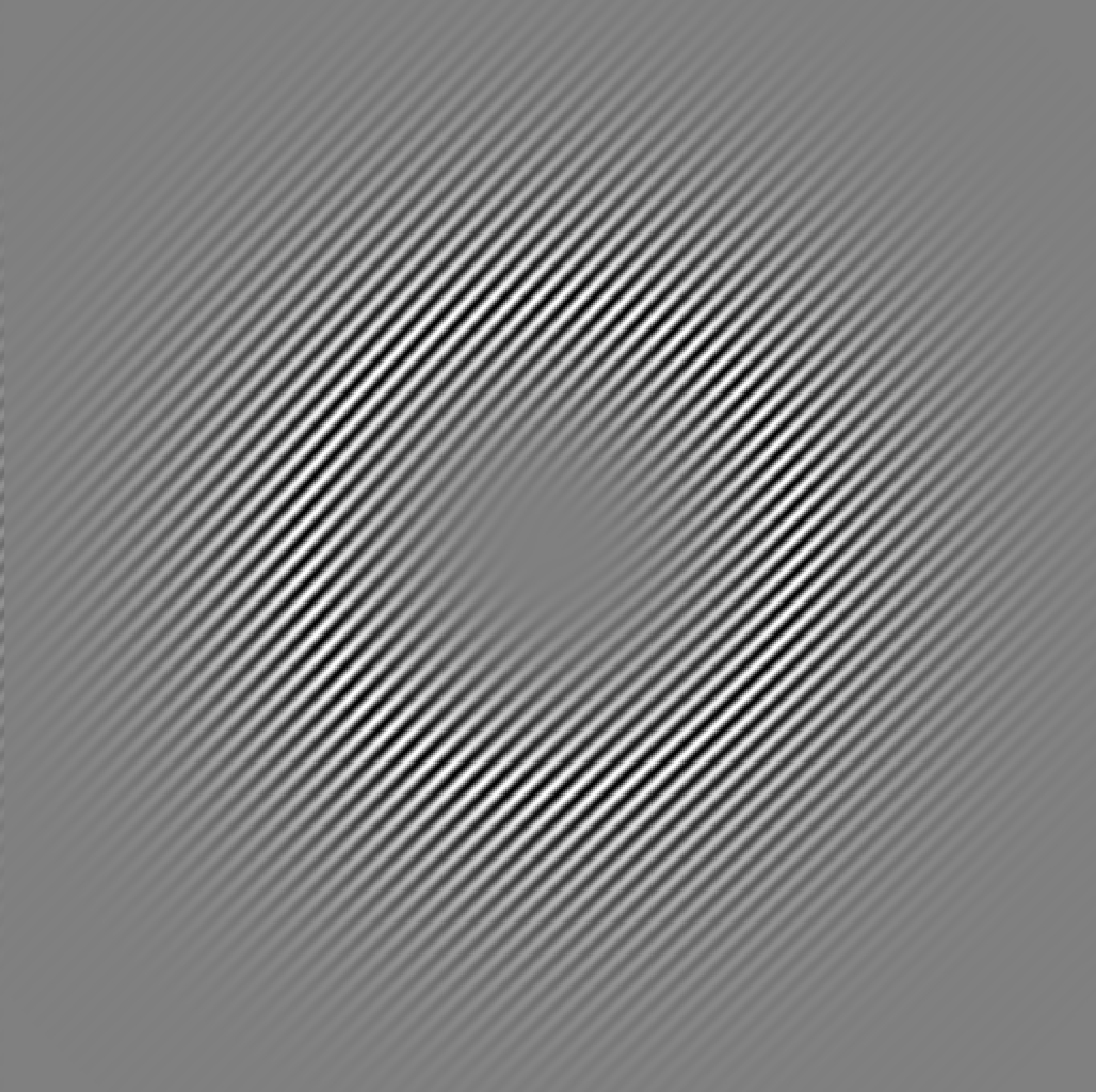

Supplement: Supplementary Information [file srep24463-s1.pdf]
